# Involvement of β- and γ-actin isoforms in actin cytoskeleton organization and migration abilities of bleb-forming human colon cancer cells

**DOI:** 10.1371/journal.pone.0173709

**Published:** 2017-03-23

**Authors:** Aleksandra Simiczyjew, Antonina Joanna Mazur, Ewelina Dratkiewicz, Dorota Nowak

**Affiliations:** Department of Cell Pathology, Faculty of Biotechnology, University of Wroclaw, Joliot-Curie 14a, Wroclaw, Poland; CIMA, SPAIN

## Abstract

Amoeboid movement is characteristic for rounded cells, which do not form strong adhesion contacts with the ECM and use blebs as migratory protrusions. It is well known that actin is the main component of mature forms of these structures, but the exact role fulfilled by non-muscle actin isoforms β- and γ- in bleb formation and migration of these cells is still not fully understood. The aim of this study was to establish the role of β- and γ-actin in migration of bleb-forming cancer cells using isoform-specific antibodies and expression of fluorescently tagged actin isoforms. We observed, after staining with monoclonal antibodies, that both actins are present in these cells in the form of a cortical ring as well as in the area of blebs. Additionally, using simultaneous expression of differentially tagged β- and γ-actin in cells, we observed that the actin isoforms are present together in a single bleb. They were involved during bleb expansion as well as retraction. Also present in the area of these protrusions formed by both isoforms were the bleb markers–ezrin and myosin II. The overexpression of β- or γ-actin led to actin cytoskeletal rearrangement followed by the growth of migration and invasion abilities of examined human colon cancer cells, LS174T line. In summary these data prove that both actin isoforms have an impact on motility of bleb-forming cancer cells. Moreover, we conclude that monoclonal antibodies directed against actin isoforms in combination with the tagged actins are good tools to study their role in important biological processes.

## Introduction

Actin is an abundant protein which is essential for proper cell functioning. It takes part in many physiological processes including cell motility, signal transduction, maintenance of cell shape, ring formation during cytokinesis, cell adhesion, transcription and muscle contraction [1]. There are six actin isoforms present in vertebrates. Among them are β- and γ-non-muscle, α-skeletal, α-cardiac, and α-and γ-smooth muscle isoactins [2]. The α-actins as well as γ-smooth muscle actin are present typically within muscle tissue, whereas non-muscle β- and γ-actin isoforms, essential for cell survival, are present in almost all cell types [3]. Actin isoforms are encoded by separate genes, but the primary structure of these proteins is very similar due to high homology among their nucleotide sequences [4,5]. The β- and γ-actin isoforms differ only by four amino acids present in the N-terminus part of the polypeptide chain. Within the β-actin structure Asp-Asp-Asp tripeptide is present at positions 2, 3, 4 and Val at position 10, whereas γ-actin contains Glu-Glu-Glu and Ile in the same positions of the polypeptide chain [2]. The β- to γ-actin ratio is distinct depending on the cell type [4,6]. In most cells the β- to γ-actin level ratio is approximately 2:1 [7,8]. Levels of actin isoforms often change in cells under pathological conditions [6,9–16].

It was also shown that β- and γ-actins in the form of both protein and mRNA are located in different cytoplasmic areas [5,17,18]. β-actin was observed in protrusive structures responsible for cell migration—lamella, pseudopod and at the tips and edges of regions involved in active movement. This isoform is also essential during intravasation of cancer cells through the vessel wall [5,13,19]. Presence of γ-actin was more often detected in the stress fibers, protrusions which are involved in regulation of cell shape and differentiation [5,20,21]. In conflicting reports Dugina et al. observed β-actin mainly in stress fibers and at cell-cell contacts, while γ-actin was observed within the leading edge. The authors postulated that β-actin is essential for cell attachment and contraction, whereas γ-actin is involved in cell motility [22,23]. It is well known that they play unique roles in many physiological processes including regulation of meiosis [24] and permeability of epithelial junctions [25], but due to the above-described controversies, the role of non-muscle β- and γ-actin isoforms in cell migration is still being examined. There were conducted some studies based on both actin isoforms’ silencing, but either only one isoform was knocked down [26,27] or overexpressed [19], or the studies focused on normal cells [22,28], which did not provide a clear answer to the question of their functional diversification in cancer cells. Recently, Dugina et al. suggested that β-actin plays the role of a tumor suppressor through inhibition of cancer cells growth and invasion, while γ-actin supports oncogenic progression [23]. Our previous results, obtained on mesenchymally migrating cancer cells overexpressing actin isoforms, showed that both actin isoforms are engaged in migration of these cells [29] and that both of them are equally involved in formation of active invadopodia [30]. We decided to overexpress β- and γ-actin, not to silence their expression, because of the quite low efficiency of this latter process indicated by others [22,31].

It is possible that the role fulfilled by non-muscle actin isoforms in cell movement is dependent on the type of migration. To obtain a more complex picture of the roles of non-muscle actin isoforms in cancer cell migration we decided to focus on the role of β- and γ-actin in migration of colon cancer cell lines LS174T and EB3, representing the bleb-forming cell mode of motility [32], and to study its effects on cell migration and invasion capacities. This type of migration was previously observed in circulating stem cells, leukocytes as well as cancer cells [33–36]. Cells moving in this way are round, loosely attached to the extracellular matrix (ECM), do not form stress fibers, but are able to squeeze through gaps in the ECM using bleb-like protrusions [33,35,37]. These very dynamic migratory blebs are formed on the leading edge of the cell, which moves forward due to contractions occurring at the opposite edge. Blebbing is stimulated by extracellular triggers [38,39], which cause destabilization of the cortical actin meshwork. It leads to formation of a plasma membrane protrusion promoted by the cytoplasmic hydrostatic pressure. The expanding bleb is not connected to the actin cortex but is coated by proteins of the ERM family such as ezrin. Then actin polymerizes in the form of bleb cortex, myosin II is recruited to the bleb lumen and RhoA-ROCK is activated, which generates the contractility necessary for retraction of the bleb [40–42]. In most cell types this mode of movement is described as much faster than the mesenchymal one [43]. Understanding the mechanism underlying bleb-forming cells’ migration is very important, because this type of movement is often used by invasive cancer cells, e.g. breast cancer, lymphoma, small cell lung cancer, prostate cancer melanoma and Walker carcinosarcoma cells [35,36,43–48].

## Materials and methods

### Materials

Mouse monoclonal anti-β- and anti-γ-actin antibodies directed against epitopes present on the N-terminus of β- or γ-actin were purchased from Sigma-Aldrich (clone AC-15 or clone 2–2.1.14.17, respectively) or from AbD Serotec (clone 4C2 or clone 2A3 respectively). Mouse monoclonal anti-GFP antibodies were obtained from Santa Cruz Biotechnology. Phalloidin conjugated with Alexa Fluor 568 and DNase I conjugated with Alexa Fluor 594 (detecting unpolymerized actin) were purchased from Invitrogen. Rabbit anti-myosin II antibodies as well as mouse monoclonal antibodies against ezrin were purchased from Sigma-Aldrich. Secondary anti-rabbit or anti-mouse antibodies conjugated with Alexa Fluor 488 or Alexa Fluor 568 were obtained from Invitrogen, while anti-mouse HRP-linked antibodies were from Cell Signaling. Fetal bovine serum (FBS), glutamine, penicillin/streptomycin, trypsin, alpha-MEM media and Lipofectamine 2000 were obtained from Invitrogen. DNase I from bovine pancreas and DNA from calf thymus were purchased from Sigma-Aldrich. Collagen type I was purchased from Corning, while epidermal growth factor (EGF) was obtained from BD Biosciences. Dako fluorescent mounting medium was obtained from Dako. All other reagents were classified as analytical grade reagents.

### Cell culture

The human colon adenocarcinoma cell line LS174T was obtained from ATCC (ATCC CL 188). EB3 cell line was obtained from the Institute of Immunology and Experimental Therapy, Polish Academy of Sciences in Wroclaw [49,50]. The cells were cultured in αMEM medium containing 10% FBS, the antibiotics—streptomycin (100 μg/ml) and penicillin (100 U/ml) and 2 mM glutamine. Cells were grown in tissue culture flasks (Sarstedt) at 37°C in 5% CO_2_/95% humidified air and passaged using 0.25% trypsin/0.05% EDTA solution twice a week.

### Actin constructs and transfection procedure

pAcGFP-C1 β- and γ-actin constructs were prepared as described in detail previously [29]. Briefly, they were generated by cloning the cDNA encoding cytoplasmic human β- or γ-actin, respectively, with their 3’-UTRs (untranslated regions), into the pAcGFP-C1 plasmid (Clontech), leading to constructs whose expression resulted in fusion proteins in which the AcGFP (*Aequorea coerulescens* Green Fluorescent Protein) moiety is at the N-terminal end of the actin polypeptide chain. Additionally, we prepared constructs whose expression resulted in fusion proteins in which the mCherry moiety was at the N-terminal end of the actin polypeptide chain. An exact description of the procedure was described in our previous articles by Simiczyjew et al. [29,30]. Lipofectamine 2000 was used to transfect the cells with pAcGFP-C1 or pmCherry encoding human β- or γ-actin, or the empty (pAcGFP-C1 or pmCherry) plasmid according to the manufacturer’s protocol. 24 h after transfection cells were used for further experiments. Cells transfected with the empty pAcGFP-C1 plasmid and thus expressing AcGFP constituted control cells. Expression of cytoplasmic actin isoforms in all transfected cells was monitored by real-time PCR and Western blotting methods.

### qRT-PCR analysis

qRT-PCR analysis was conducted as described previously by Simiczyjew et al. [29]. Total RNA was extracted using the NucleoSpin RNA II Kit (Macherey-Nagel) and the reverse transcription reaction was performed using 0.5 μg of RNA and the High Capacity cDNA Reverse Transcription Kit (Applied Biosystems) following the manufacturer's instructions.

Transcribed cDNA was used for the subsequent qRT-PCR (quantitative reverse transcription polymerase chain reaction) with specific primers. Real-Time 2xHS-PCR Master Mix SYBR A (A&A Biotechnology) was used for qRT-PCR reactions, which were carried out in a Roche LightCycler 2.0 (Roche). For quantification the samples were normalized against the expression of GAPDH, TUBA1C, HSP90AA1 and 18S mRNA. It was done since e.g. GAPDH was not stably expressed in LS174T cells. All experiments were done in triplicate. The exact procedure, primers used and all necessary controls were described in our previous paper [29].

### Isolation of cytosolic fractions

Cytosolic fractions were prepared as described in detail previously [29]. Briefly, cells were homogenized and the cytosolic fraction was isolated as described previously by Malicka-Błaszkiewicz and Roth [51]. Cells transiently overexpressing AcGFP, AcGFP-β- or γ-actin were washed with PBS, scraped with a cell scraper, and suspended in freshly prepared monomeric actin stabilizing buffer A. Then cells were centrifuged (1000 x *g*, 3 minutes, 4°C) and homogenized with 3 volumes of freshly made buffer A in a Dounce homogenizer. Homogenates were ultracentrifuged at 105 000 x *g* for 1 h at 4°C. The supernatant was used as the cytosolic fraction. All experiments were done in triplicate.

### Isolation of cellular extracts

Cellular extracts for Western blotting analysis were prepared as described previously [29]. Briefly, the LS174T cells were lysed with cytoskeletal-bound protein extraction buffer on ice. Then cells were threefold frozen-thawed and centrifuged at 10 000 x *g* for 10 minutes at 4°C.

### Western blot analysis

The standard Bradford procedure was used to determine the protein concentration in cellular extracts [52]. Samples were separated by SDS-PAGE electrophoresis according to the procedure described by Laemmli [53] and then transferred to nitrocellulose sheets, according to Towbin et al. [54]. For AcGFP-actin (70 kDa) and AcGFP (27 kDa) identification mouse anti-GFP antibodies were applied. Monoclonal mouse anti- β-actin antibodies and monoclonal mouse anti-γ-actin antibodies were used for endogenous β-actin (43 kDa) and endogenous γ-actin (43 kDa) identification. As an internal loading control β-tubulin, recognized by monoclonal mouse antibodies directed against β-tubulin, was used. Next goat anti-mouse antibodies conjugated to HRP were applied. Immunoblots were visualized using the Western blotting Luminol Reagent (Santa Cruz Biotechnology). All experiments were done in triplicate.

### Confocal microscopy

Actin cytoskeleton organization in examined cells was observed under a confocal laser scanning microscope (Olympus FV 500), after staining conducted as described in detail previously [29,30]. Briefly, cells were seeded on sterile coverslips and grown for 24 h. Then, when indicated, cells were transfected and 24 h later fixed with 4% formaldehyde and permeabilized with 0.1% Triton X-100 or with methanol in the case of staining with antibodies recognizing β- or γ-actin. After blocking with 1% bovine serum albumin, to visualize non-muscle actins, monoclonal anti-β-actin and anti-γ-actin antibodies were applied, followed by Alexa Fluor 568-conjugated anti-mouse secondary antibodies. Primary anti-ezrin and anti-myosin II antibodies and then secondary anti-mouse or anti-rabbit antibodies conjugated with Alexa Fluor 488 or Alexa Fluor 568 were used to observe ezrin and myosin II. Alexa Fluor 568-labeled phalloidin was applied to visualize actin filaments and DNase I conjugated with Alexa Fluor 594 to stain monomeric actin. The overexpression of β-actin and γ-actin was observed as fluorescence of the fusion protein (AcGFP-β-actin and AcGFP-γ-actin). All experiments were done in triplicate. Colocalization parameters were measured by ImageJ software with the help of JACoP plugin [55] as an average value measured in 15 randomly selected cells. Colocalization of proteins was determined using the Pearson’s coefficient, a method to measure the degree of colocalization of objects in confocal dual-colour images. Its value can range from 1 to -1, with 1 standing for complete positive correlation and -1 for a negative correlation, with zero standing for no correlation.

### Time-lapse microscopy analysis

For live-cell imaging, LS174T cells were transfected with pACGFP-β-actin or pAcGFP-γ-actin plasmids. Fluorescence microscopy was carried out at 37°C and 5% CO_2_, using a Delta Vision Elite inverted microscope equipped with a 100x oil-immersion objective, ultimate focus and Olympus IX71 camera. Images were acquired every 20 seconds over a span of 20 minutes using EGFP filter set. Images were analyzed using FIJI software.

### *In situ* proximity ligation assay

The *in situ* proximity ligation assay (PLA) was conducted as described in detail previously [30]. This method is based on the idea that if two proteins are in proximity, secondary antibodies with attached oligonucleotides are close enough to form a circular DNA structure, which can be amplified after ligation using polymerase solution. In the amplification solution, a fluorophore-tagged DNA probe is also present. Its sequence is complementary to the one being amplified; that is why the probe can bind to it, which allows one to visualize the close proximity between two investigated proteins [56]. PLA experiments were performed using Duolink reagents according to the manufacturer’s protocol (Sigma-Aldrich). Briefly, LS174T cells were fixed with 4% formaldehyde and incubated for 5 min with ice cold methanol. Nonspecific binding sites were blocked with 1% BSA. Cells were incubated with an appropriate pairs of mouse and rabbit primary antibodies (mouse anti-β-actin/rabbit anti-myosin II; mouse anti-γ-actin/ rabbit anti-myosin II). Then cells were incubated with anti-mouse PLA probe MINUS and anti-rabbit PLA probe PLUS (both diluted 1:5 in 1% BSA for 1 h at 37°C) with extensive washing between these steps. Cells were washed with buffer A (see manufacturer’s protocol), incubated with ligation solution (30 min, 37°C), again washed with buffer A and incubated with amplification solution (100 min, 37°C). Ultimately, cells were washed with buffer B (see manufacturer’s protocol), mounted onto glass coverslips using Duolink *in situ* mounting medium with DAPI, and examined with a ZEISS LSM 510 confocal microscope.

### Evaluation of actin polymerization state

Actin polymerization state was evaluated by measuring the inhibition of DNase I from bovine pancreas under standard assay conditions [51]. The applied procedure was described previously [29]. The concentration of monomeric (G) actin was estimated by DNase I inhibition, directly in the cytosolic fraction of the cells. Total (T) actin content was measured after dilution of the samples with G actin stabilizing buffer (buffer A). For the measurement of maximal inhibition a specific dilution below the critical actin concentration had to be applied to completely depolymerize filamentous (F) actin. The amount of F actin was calculated by subtracting the amount of G actin from the total actin (F = T–G). The state of actin polymerization was defined by the F actin to G actin ratio (F:G). One unit of DNase I inhibitor (actin) is the amount that reduces the activity of 20 ng of DNase I by 10% under standard assay conditions [51,57]. Actin concentration was expressed in units of DNase I inhibitor per mg of sample protein. The experiments were performed three times, each as an independent experiment. Each independent experiment consisted of three measurements/probes.

### Migration assay

The migration assay was conducted as described previously [29]. Briefly, migration tests were performed using Transwell filters. Control cells (transfected with pAcGFP-C1 plasmid) and cells overexpressing β- or γ-actin, after starvation, were seeded onto Transwell inserts in medium without FBS and EGF. The lower compartment of the filter was filled with 500 μl of medium containing 20% FBS and 5 nM EGF. After 24 h, non-migrating cells from the upper side of the filters were removed. Cells which migrated through the membrane were fixed with 4% formaldehyde, stained with Hoechst 33342 and counted under a fluorescent microscope. The results are presented as a relative migration factor (%), where control cells which migrated through the Transwell filters are presented as 100%. The experiments were performed three times. Each independent experiment consisted of three measurements.

### Invasion assay

The exact procedure of the invasion assay was described previously [29]. Tests were performed using the Boyden chamber assay. Transfected cells, after starvation, were seeded onto Transwell filters coated with Collagen type I. Medium containing 20% FBS and 5 nM EGF was used in the lower compartment as a chemoattractant. After 24 h non-migrating cells on the upper side of the filters were removed. Cells which migrated through the membrane were fixed with 4% formaldehyde, stained with Hoechst 33342 and counted under a fluorescent microscope. The results are presented as the relative invasion factor (%), where control cells which migrated through the Transwell filters are presented as 100%.

### Statistical analysis

All data are shown as means ± standard deviations (S.D.), and their statistical significance was evaluated with the two-tailed, unpaired Student's t-test or via one-way ANOVA followed by the Tukey post hoc test. The level of significance was set at p<0.05 or p<0.01.

## Results

### Localization of actin isoforms within bleb-forming colon cancer cells

**Fig 1:**
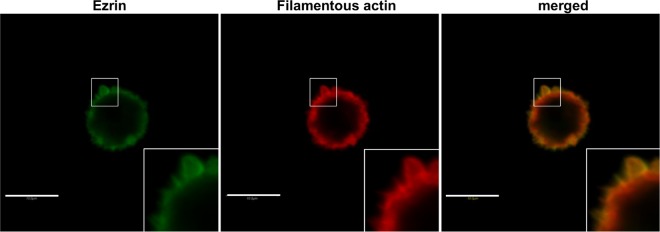
Bleb-like protrusions in LS174T colon cancer cells with rounded morphology. Cells were plated onto coverslips. After fixation with 4% formaldehyde, cells were labeled to visualize filamentous actin (red) and ezrin (green). Merged image is shown on the right picture. Enlargements of the boxed, bleb-rich area are shown as insets. Scale bar: 10 μm

**Fig 2:**
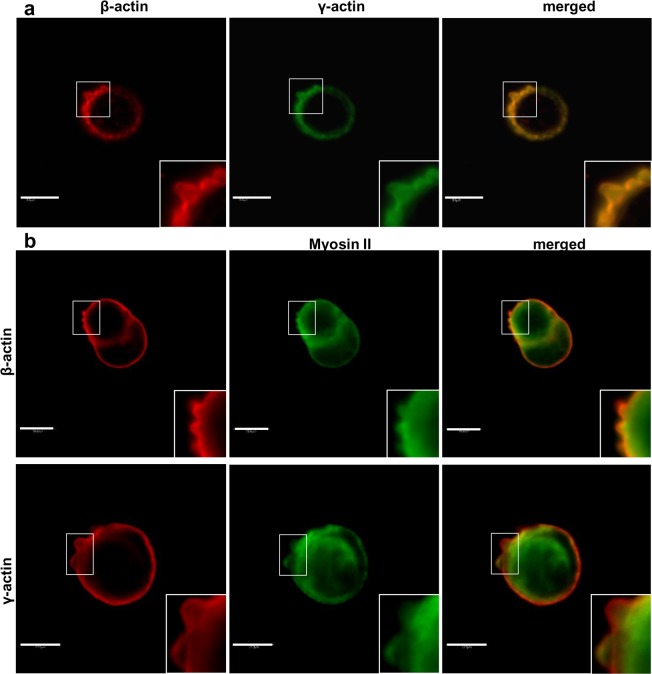
Subcellular localization of β- and γ-actin as well as myosin II in LS174T cells. Cells were plated onto coverslips. (a,b) After fixation with 4% formaldehyde, cells were labeled to visualize β-actin and γ-actin (using antibodies from Sigma Aldrich or AbD Setorec) (a) as well as their colocalization with myosin II (b). Merged images are shown in the right pictures. Enlargements of the boxed, bleb-rich area are shown as insets. Scale bar: 10 μm.

**Fig 3:**
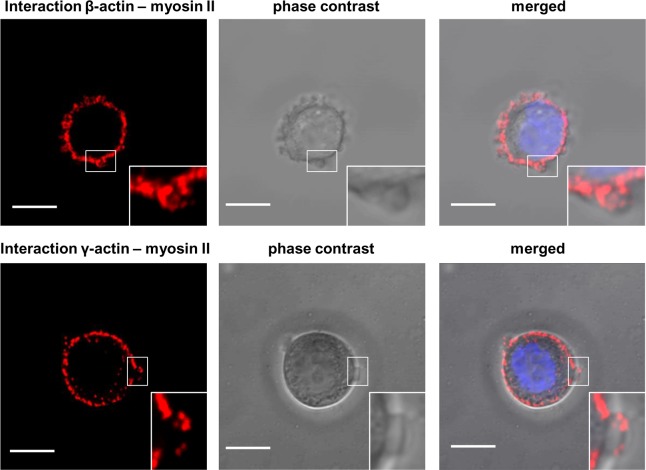
Visualization of interactions between β- or γ-actin and myosin II using in situ PLA. The proximity ligation assay between endogenous β- or γ-actin and myosin II was performed as described in Materials and Methods. Each red spot in the left panel represents a single interaction between endogenous β- or γ-actin and myosin II. The middle panel presents images of LS174T cells in the phase contrast. Merged images (interaction of proteins, phase contrast image, nucleus stained with DAPI) are shown on the right panel. Scale bar: 10 μm.

We have previously shown that both actin isoforms, β and γ, are present within multiple F-actin-rich membrane protrusions formed by mesenchymally migrating cells [29,30]. Now we decided to verify involvement of these actins in cytoskeleton organization and migration of bleb-forming cancer cells. Human colon adenocarcinoma LS174T cells, migrating in a bleb-dependent manner [32], were used as a cell model in conducted experiments. At first we checked whether blebs formed by the cells did not result from the fact that the examined cells undergo apoptosis. Using apoptotic markers (annexin V, nucleus morphology, propidium iodide) we excluded this possibility (data not shown). The actin cytoskeleton of these cells is visible as a cortical ring under the cellular membrane with bleb-like protrusions containing filamentous actin as well as ezrin (Fig 1), which links the actin cytoskeleton to the cell membrane and is essential for bleb formation and can be treated as a migratory bleb marker [40,58,59]. Results presented in Fig 2A, obtained by using different monoclonal antibodies specific for each actin isoform, show that both β- and γ-actin colocalize under the cell membrane of examined cells, as well as within migratory blebs (S1 Table). In the area of these structures both actin isoforms colocalize with myosin II (Fig 2B)—which is crucial for bleb retraction [58–60]. Myosin is localized more centrally in blebs as well as at the base of these protrusions, while β- and γ-actin are located more peripherally (Fig 2B). Due to a previous report that β- and γ- non-muscle actin isoforms are able to differentially regulate nonsarcomeric myosins’ activity [61], we decided to verify the interaction between these actin isoforms and myosin II in bleb-forming cancer cells. The PLA (In Situ Proximity Ligation Assay) method was applied to investigate the proximity of these proteins in examined cells. This method allows one to visualize close proximity (up to 40 nm) between two proteins. We detected close proximity between the β- and γ-actin isoforms and myosin II in the submembranous area as well as within blebs (Fig 3). However, we were not able to observe statistically significant differences in interaction between the pairs β-actin/myosin II versus γ-actin/myosin II.

### Influence of overexpression of β- or γ-actin isoform on actin cytoskeleton organization and actin polymerization state in bleb-forming colon cancer cells

**Fig 4:**
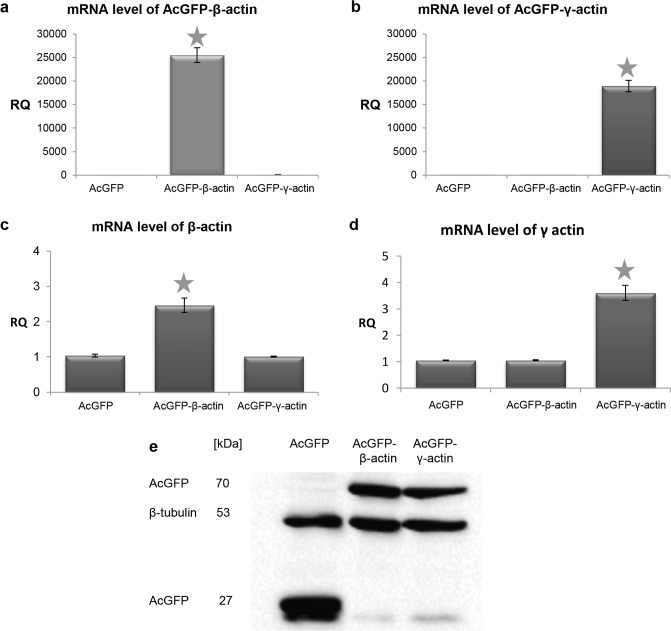
Expression of actin isoforms on mRNA and protein levels in LS174T cells transiently transfected with plasmids coding for β- or γ-actin. The mRNA levels of AcGFP-β-actin (a) or AcGFP-γ-actin (b) and total β- (c) or γ-actin (d) were measured by qRT-PCR. Asterisks indicate values statistically different from those obtained for the control cells, transfected with the pAcGFP-C1 plasmid. The significance level was set at p ≤ 0.01 in one-way ANOVA followed by Tukey post hoc test. The data were obtained from three independent experiments. (e) Western blot analysis of AcGFP. A representative immunoblot identifying AcGFP and fusion proteins in cellular extracts of control cells (transfected with pAcGFP-C1) and cells overexpressing AcGFP tagged β- or γ-actin. Used antibodies: monoclonal mouse antibodies directed against β-tubulin, mouse monoclonal antibodies directed against GFP.

In order to confirm previous results we decided to additionally overexpress both actin isoforms in examined cells. We prepared plasmids containing cDNAs of β- and γ-actins with their 3’-UTRs as well as the sequence encoding AcGFP (*Aequorea coerulescens* Green Fluorescent Protein) as described previously by Simiczyjew et al. [29]. We preserved 3’-UTRs, because it was shown previously that these regions of mRNA are essential for appropriate localization of at least β-actin in the cell [62–64]. The fluorescent tag (AcGFP) is fused to the actin N-terminus, because tagging actin on its C-terminus can result in disruption of the actin folding process [65,66]. After transfection the amount of mRNA encoding fusion proteins—AcGFP-actins—was measured by qRT-PCR. The exact procedure (including primer sequences) was described by us previously [29]. At first we generated stable clones of LS174T cells, but unfortunately, as in the case of mesenchymally migrating cells [29], also this time the signal for AcGFP-actins obtained during Western blot analysis was very low (data not shown). It indicates that the level of β- or γ-actin within cells is strictly regulated. For this reason we decided to transiently transfect cells with plasmids encoding tagged β- or γ-actin. This method was much more efficient, and for this reason cells transiently overexpressing non-muscle actin isoforms were chosen for further experiments. qRT-PCR (Fig 4A–4D) and Western blot analysis (Fig 4E) confirmed the elevated level of expression of both examined actin isoforms in transfected cells in relation to control cells, transfected only with the empty pAcGFP-C1 plasmid. Because N-terminus of actins was tagged with AcGFP, it was impossible to use specific antibodies directed against individual actin isoforms. All antibodies which allow one to detect β- or γ-actin are directed against the epitope situated at the N-terminus of these proteins, since they differ only by four amino acids present in this part of both actins. Therefore, to detect tagged actin isoforms in cell extracts we used monoclonal antibodies recognizing GFP (Fig 4E). Simultaneously, we verified that endogenous non-muscle actin isoforms level does not change after transfection (S1 Fig).

**Fig 5:**
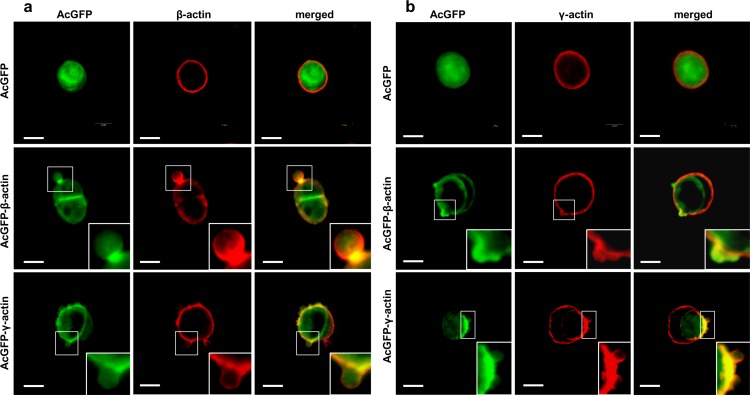
**Subcellular distribution of β- (a) and γ- (b) actin in LS174T cells with increased level of actin isoforms.** Lower rows in panels a and b show representative LS174T cells overexpressing β- or γ-actin, respectively. Left panel: AcGFP fluorescence (green), middle panel: endogenous β- or γ-actin stained with mouse anti-β- or anti-γ-actin antibody (red). Merged images are shown on the right panel. Enlargements of the boxed, bleb-rich area are shown as insets. Scale bar: 10 μm.

**Fig 6:**
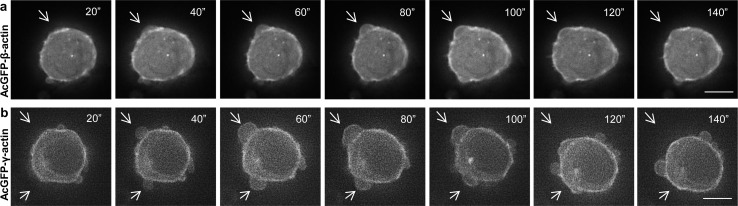
**Time-lapse sequences of LS174T cells forming blebs after pAcGFP-β-actin (a) and pAcGFP-γ-actin (b) transfection.** Transfected cells were observed using time lapse fluorescent microscope. Images were acquired every 20 seconds for 20 minutes using EGFP filter set. Time is expressed in sec (“). White arrows indicate expanding and retracting blebs. Scale bar represents 5μm.

In the next step LS174T cells overexpressing AcGFP-actins were analyzed by scanning confocal microscopy. All results obtained for these cells were compared to control cells, expressing only AcGFP. To visualize intracellular localization of endogenous β- or γ-actin we used monoclonal antibodies directed against β- or γ-actin, respectively. Exogenous actin isoforms, present in transfected cells, were observed using fluorescence of green fluorescent protein (AcGFP). Both overexpressed actins, β and γ, were present as a cortical ring in the submembranous region of the cell body and colocalized strictly, in contrast to AcGFP in control cells, with both endogenous actins (Fig 5A and 5B). Both endogenous and exogenous actins were also present within bleb-like protrusions (Fig 5, insets). It indicates that exogenous proteins—AcGFP-conjugated actins play a similar role as endogenous actin in LS174T cells. We also noted that cells overexpressing β-actin or γ-actin have a higher ability to form these protrusions than control cells (Fig 5). Additionally, using time-lapse microscopy, we indicated that both actin isoforms are present within blebs during expansion as well as retraction. These results are illustrated in Fig 6 and also in S1 Movie and S2 Movie.

**Fig 7:**
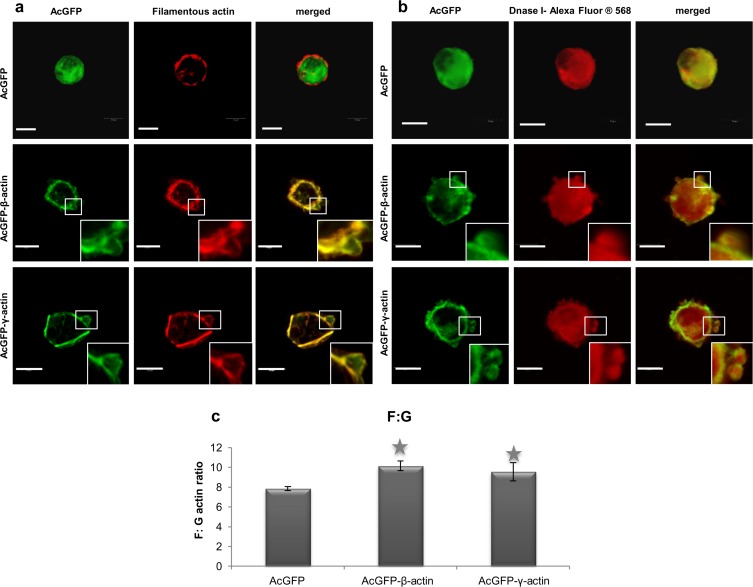
Actin organization in LS174T colon cancer cells overexpressing β- and γ-actin isoforms. (a) Filamentous actin organization in examined cells with increased level of β- or γ-actin. Confocal images showing cells expressing actin isoform β- or γ- encoded by pAcGFP-C1 expression vector were compared to cells transfected with an empty vector pAcGFP-C1. Left panel: AcGFP fluorescence (green). Middle panel: filamentous actin visualized by staining with AlexaFluor 568-conjugated phalloidin (red). Merged images are shown on the right panel. Scale bar: 10 μm. Enlargements of the boxed, bleb-rich area are shown as insets. (b) Subcellular localization of monomeric actin in cells overexpressing β- or γ-actin. β- and γ-actin were encoded by pAcGFP-C1 expression vector. Left panel: AcGFP (green). Middle panel: monomeric actin visualized by staining with DNase I conjugated with Alexa Fluor 594 (red). Merged images are shown on the right panel. Scale bar: 10 μm. (c) Actin polymerization state in colon cancer cells overexpressing β- or γ-actin. Asterisks indicate values statistically different from those obtained for the control cells, transfected with pAcGFP-C1 plasmid. The significance level was set at p ≤ 0.01 in one-way ANOVA followed by Tukey post hoc test. The data were obtained from three independent experiments.

Then we stained transfected cells with phalloidin-Alexa Fluor 568 and analyzed the distribution of filamentous actin (F actin) within them (Fig 7A). In LS174T adenocarcinoma cells expressing pAcGFP-β-actin or pAcGFP-γ-actin, actin was present in filamentous form under the cell membrane, and also on the edge of bleb-like protrusions (Fig 7A, insets). Afterwards the cells, control and transfected with pAcGFP-β-actin or pAcGFP-γ-actin, were stained with DNase I conjugated with Alexa Fluor 594. It binds much more strongly to monomeric actin (G actin) than to filamentous actin, and this feature allowed us to analyze distribution of G actin in these cells (Fig 7B). In cells overexpressing β- or γ-actin G actin was localized in areas of bleb formation and in the whole cell body (Fig 7B).

Due to the fact, that regulation of actin polymerization plays an essential role in the process of cancer cell migration and previous studies showed the existence of a correlation between the metastatic potential of human cancer cells and the state of actin polymerization [67–70], we decided to determine the filamentous to monomeric actin ratio (F:G) in examined cells (Fig 7C). The applied method, described in the Materials and Methods section, allows quantitative calculation of the relation between the amount of monomeric and non-monomeric actin within cells and can be treated as a complement to qualitative confocal images. We observed a statistically significant increase in the F:G actin ratio in AcGFP-β-actin and AcGFP-γ-actin overexpressing cells in comparison to control cells (Fig 7C).

Based on data obtained by F and G actin staining within cells and actin polymerization ratio analysis we conclude that overexpressed β- and γ-actin are present in cells mainly in filamentous form.

### The role of β- and γ-non-muscle actin isoforms in bleb formation and migration of colon cancer cells

**Fig 8:**
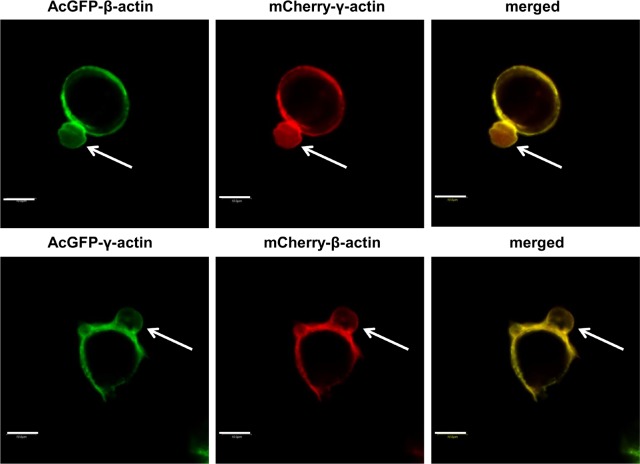
Exogenous β- and γ-actin are simultaneously present within blebs formed by transfected LS174T cells. Confocal images showing cells overexpressing AcGFP-γ-actin and mCherry-β-actin or mCherry-γ-actin and AcGFP-β-actin. Left panel: AcGFP fluorescence. Middle panel: mCherry fluorescence. Merged images are shown on the right panel. Blebs are indicated by white arrows. Scale bar: 10 μm.

Our next goal was simultaneous transfection of LS174T cells with two plasmids: pAcGFP-γ-actin and pmCherry-β-actin (or vice versa). Its aim was further confirmation that both examined actin isoforms are components of blebs. This method allowed us to observe the distribution of β- and γ-actin within a single cell and within one bleb. The overexpressed actins colocalized in the submebranous area as well as within migratory blebs (Fig 8, S1 Table). We also observed that these cells exhibited a greater ability to form large blebs than control cells (transfected with empty plasmids). This result confirms data obtained previously on non-transfected cells, stained with antibodies directed against β- and γ-actin (Fig 2A). In conclusion, according to our knowledge this is the first report indicating that β- and γ-actin are simultaneously present within bleb-like structures.

**Fig 9:**
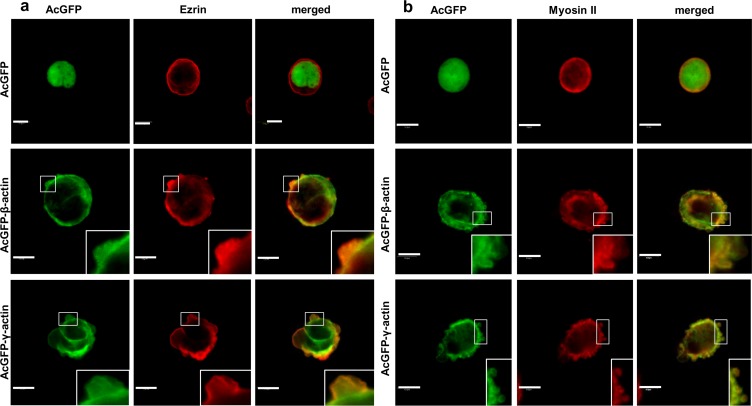
Co-localization of exogenous cytoplasmic actin isoform with bleb markers. Confocal images showing LS174T cells expressing actin isoform β- or γ- encoded by pAcGFP-C1 expression vector were compared to cells transfected with the empty vector pAcGFP-C1. (a) Left panel: AcGFP fluorescence, middle panel: ezrin stained with mouse monoclonal antibodies. Merged images are shown on the right panel. (b) Left panel: AcGFP fluorescence, middle panel: myosin II stained with rabbit polyclonal antibodies. Merged images are shown on the right panel. Enlargements of the boxed, bleb-rich area are shown as insets. Scale bar: 10 μm.

In blebs formed by LS174T cells which are positive for AcGFP-β-actin or AcGFP-γ-actin, we also observed colocalization of both exogenous actins with the bleb marker—ezrin (Fig 9A, S1 Table) and occurrence of myosin II in the central area of these protrusions, while both exogenous actin isoforms were observed a slightly more peripherally (Fig 9B, S1 Table). Cells with AcGFP expression were used as a control. The similar staining patterns as in Figs 1 and 2B indicate that exogenous actins “behave” similarly as the endogenous ones.

**Fig 10:**
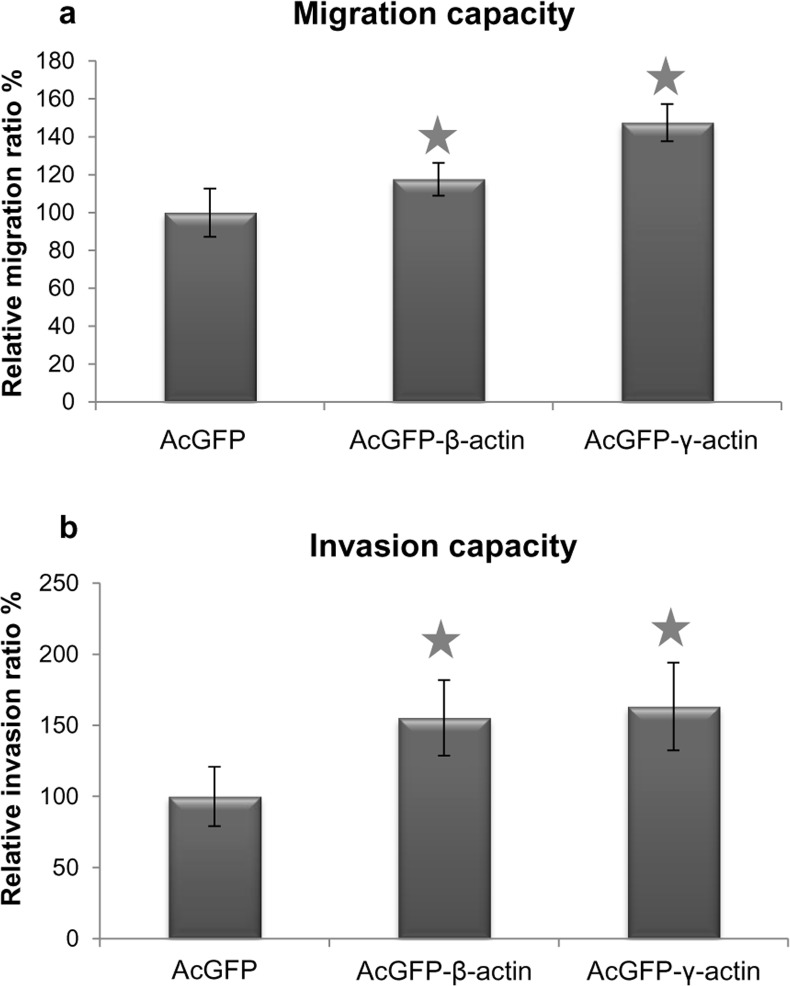
**Migration (a) and invasion (b) capacities of LS174T cells overexpressing β- or γ-actin isoform.** Results expressed as the mean ± SD are representative for at least three independent experiments. Migration and invasion in control cells are presented as 100%. Asterisks indicate values statistically different from those obtained for the control cells, transfected with pAcGFP-C1 plasmid. The significance level was set at p ≤ 0.05 in Student’s t-test.

Then a migration assay was conducted to verify whether the elevated expression of both actin isoforms has an influence the migration and invasion capacities of colon cancer cells. LS174T cells transfected with plasmids encoding AcGFP-β-actin or AcGFP-γ-actin showed a significant increase in migration capacity in comparison to control cells (Fig 10A). The invasion assay, in which cells invade through a Transwell filter coated with collagen type I which imitates 3D conditions, gave similar outcomes as results from the migration assay (Fig 10B).

Finally, we decided to conduct analogous experiments on the other bleb-forming human colon cancer cell line–EB3 to verify our observations for LS174T. Obtained outcomes corroborated those results (S2, S3, S4, S5 and S6 Figs).

## Discussion

The migration of single cancer cells can be divided into amoeboid and mesenchymal types of movement. Cells migrating in mesenchymal mode are elongated, form strong focal attachment to the ECM, and use proteases to digest matrix and invade through the tissues. Amoeboid movement is characteristic for rounded cells, which do not form strong adhesion contacts with the ECM and use blebs to squeeze through gaps within the matrix. Cancer cells are able to use both types of migration as well as change their mode of movement in response to external conditions [33,38,71–73].

It is well known that the actin cytoskeleton is the main component of migratory protrusions, but the exact role fulfilled by non-muscle actin isoforms in their formation and activity is still not established. However, it was demonstrated previously, that β- and γ-actin isoforms are able to fulfill different functions, e.g. in cell motility [13,22,23,31,74,75], cell division [24], gene transcription [76], endothelial cell motility and neovessel maintenance [77] as well as in developmental processes [78,79].

The exact role of both proteins in cell migration process has been studied for many years. Some researchers tried to clarify it by observation of the effects of overexpression or silencing of β- or γ-actin [19,22,27,31,74,80], but unfortunately they were not able to establish the exact function of non-muscle actin isoforms in migration of cancer cells. The most recent research concerning the role of these actin isoforms within cancer cells was conducted by Dugina et al. [23]. The authors postulated that β-actin is a tumor suppressor, which inhibits cell growth and invasion, while γ- actin increases the oncogenic potential of selected cancer cells [23]. Our previous experiments on cancer cells representing the mesenchymal mode of movement demonstrated that both β- and γ-actin are located in the migratory protrusions, such as lamellipodia and invadopodia. Moreover, we showed that they play an equivalent role in migration of these cells [29,30].

Due to known involvement of actin also in migration of blebbing cells [32,48,81], we decided to verify the role of both β- and γ-actin in migration of bleb-forming cancer cells, using commercially available isoform-specific antibodies, as well as via expression of fluorescently-tagged actin isoforms.

Liu et al. stated that there are two distinct types of ameboidally migrating cells. Cells of the first type (A1) were round with a small leading edge. Cells of the second type (A2) had an elongated ellipsoid cell body with a large uropod and resembled a migrating neutrophil. Generally A2 cells moved significantly faster than A1 cells. The type of fast migration, A1 or A2, varied depending on the cell line under observation [82]. The examined LS174T colon cancer cells are round and form numerous blebs (Fig 1). That is why, in our opinion, these cells should be classified as A1 type.

After staining with monoclonal antibodies we observed that both actins are present in these cells in the form of a cortical ring as well as in the area of blebs (Fig 2A). Then we decided to verify our results using cells expressing exogenous actins—AcGFP-β- or pAcGFP-γ-actin. We had previously validated the functionality of the AcGFP-tagged actins for actin isoforms’ functional diversification [29]. Transfected LS174T cells also formed bleb-like membrane protrusions in which endogenous and exogenous β- or γ-actin colocalized (Fig 5). This strongly indicates, that both actin isoforms participate in the cellular motility of these cells and confirms, that fusion proteins—AcGFP-conjugated actins—possess biological activity similar to endogenous actins. We also showed that both exogenous actins are involved in bleb formation as well as retraction (Fig 6). Furthermore, using co-expression of differently tagged actin isoforms (AcGFP-β-actin/mCherry-γ-actin or mCherry-β-actin/AcGFP-γ-actin), we demonstrated that the isoforms are simultaneously present in one bleb. Additionally, these cells were able to form bigger blebs than control cells (Fig 8). This report is the first one to show simultaneous presence of both actin isoforms within these structures.

Migration and invasion processes in cancer cells are regulated by actin binding proteins. In bleb-associated migration myosin II and proteins from the ERM family—especially ezrin are involved mainly. Both of these proteins are present within a bleb at certain stages of its life cycle. Development of a bleb can be divided into three phases: initiation, growth and retraction [40,42,59]. During nucleation the membrane detaches from the cortex. Then he bleb grows by filling up with cytosol and forms a spherical protrusion devoid of filamentous actin [58,83]. Later there occurs expansion, and when it slows down ezrin is recruited to the bleb rim independently of actin. In the next stage the actin cortex appears, followed by actin-bundling proteins recruitment. Finally, the motor protein myosin II is recruited to the bleb and powers retraction [40,48,58].

In LS174T bleb-forming cells the β- and γ-actin signals colocalized with the blebs marker ezrin (Figs 1 and 9A). The role of ezrin level and localization in blebbing cells is ambiguous. Some researchers have reported, that during the bleb initiation process ezrin is enriched at the back of the cells, which leads to reduced membrane to cortex attachment at the leading edge and supports bleb formation in this part of a cell [59,60,84]. On the other hand we observed previously the presence of ezrin within the leading edge of colon cancer cells [85]. It was also found that ezrin is the linker between the actin cortex and the cell membrane within this protrusion and plays an important role in the stabilization of membrane-actin attachment in the retracting bleb [41,42]. Therefore ezrin is essential for proper bleb functioning, but not at the phase of bleb formation.

Another protein that is necessary for formation and retraction of these protrusions is myosin II. It was shown that bleb formation is critically dependent on the level of myosin contractility [59]. It also contributes to the extension of blebs by increasing intracellular pressure [86]. Myosin II recruitment to the bleb is thought to drive bleb retraction. It was found to localize within the bleb underneath the actin shell [41,42]. Our experiments, on both endogenous and exogenous actins, also demonstrated that myosin II is located in the peripheral part of blebs consisting of β- or γ-actin (Figs 2B and 9B). Similar results were obtained by Charras et al. except that there just actin was observed, without distinction between isoforms [41]. Additionally, it was reported that β- and γ-actin isoforms contribute to the modulation of non-muscle myosin-II and myosin-VII activity, and thereby to the spatial and temporal regulation of cytoskeletal dynamics [61]. We decided to verify the interaction between non-muscle actin isoforms and myosin II in examined cells using the PLA assay. This method allows one to verify that two proteins are in such close proximity (less than 40 nm), that they can interact with each other. We observed that both isoforms interact with myosin in the submembranous area, as well as within blebs formed by these cells. However, we were not able to confirm quantitative differences in these interactions (Fig 3), which may suggest that β- and γ-actin are equally involved in actomyosin contractility responsible for bleb formation and retraction.

Our data proved that both overexpressed isoforms are present in cells, mainly in filamentous form as a cortical ring under the cellular membrane (Fig 7A). Moreover, they were also present as a monomeric actin in the whole cell body as well as in cell protrusions (Fig 7B). These experiments were complemented by analysis of the actin polymerization state. They showed that in cells overexpressing both non-muscle actin isoforms the F:G actin ratio is elevated in comparison to control cells (Fig 6C). Previous studies showed a positive correlation between high actin polymerization state and the metastatic potential of human cancer cells [67–70]. Relative migration and invasion ratios are higher for β- or γ-actin overexpressing cells in relation to control cells (Fig 10). This is in line with our previous observations of mesenchymally migrating cancer cells, where the overexpression of both non-muscle actin isoforms increased migration abilities of examined cells [29]. Although we do not observe predominance of one of non-muscle actins in bleb-forming process, we do not exclude possibility that there is a diversification of function of these isoforms. Nevertheless, it is possible that there may exist differences in polymerization dynamics of both isoforms depending on Ca^2+^ /Mg^2+^ ion concentration in the cytoplasm of cancer cells [8]. Additionally, Dugina et al. proved that during tumor progression γ-actin interacts with some signaling proteins such as pErk1/2 and PP1 much stronger than β-actin [23].

In summary, our results clearly indicate that both non-muscle actin isoforms are engaged in bleb formation as well as in migration and invasion of blebbing cancer cells. The research presented here also confirms that the use of tagged-actins constructs in combination with monoclonal antibodies directed against β- and γ-actin provides a good instrument to study migratory protrusions formed by cancer cells and to clarify a possible unique role of the non-muscle actin isoforms in important biological processes.

## Supporting information

S1 Table Colocalization between selected proteins quantified using Pearson’s correlation coefficient. The Pearson’s coefficient for all examined pairs of proteins indicates positive correlation with all values above 0,5 (where 1 is absolute positive correlation, 0 means neutral interaction and -1 indicates absolute negative correlation) Results (from 15 images) are expressed as mean ± standard deviation.
(DOCX)

S1 Fig Western blot analysis of endogenous actins level in transfected LS174T cells. A representative immunoblots identifying endogenous β- or γ-actin as well as β- tubulin in cellular extracts of control cells (transfected with pAcGFP-C1) and cells overexpressing AcGFP tagged β- or γ-actin. Used antibodies: monoclonal mouse antibodies directed against: β- or γ-actin and β-tubulin.
(TIF)

S2 Fig Bleb-like protrusions in EB3 colon cancer cells with rounded morphology. Cells were plated onto coverslips. After fixation with 4% formaldehyde, cells were labeled to visualize filamentous actin (red) and ezrin (green). Merged image is shown in the right picture. Enlargements of the boxed, bleb-rich area are shown as insets. Scale bar: 5 μm
(TIF)

S3 Fig Subcellular localization of β- and γ-actin as well as myosin II in EB3 cells. Cells were plated onto coverslips. (a,b) After fixation with 4% formaldehyde, cells were labeled to visualize β-actin and γ-actin (a) as well as their colocalization with myosin II (b). Merged images are shown in the right pictures. Enlargements of the boxed, bleb-rich area are shown as insets. Scale bar: 5 μm.
(TIF)

S4 Fig **Subcellular distribution of β- (a) and γ- (b) actin in EB3 cells with increased level of actin isoforms.** Lower rows in panels a and b show representative EB3 cells overexpressing β- or γ-actin, respectively. Left panel: AcGFP fluorescence (green), middle panel: endogenous β- or γ-actin stained with mouse anti-β- or anti-γ-actin antibody (red). Merged images are shown on the right panel. Enlargements of the boxed, bleb-rich area are shown as insets. Scale bar: 5 μm.
(TIF)

S5 Fig Colocalization of exogenous cytoplasmic actin isoform with blebs markers. Confocal images showing EB3 cells expressing actin isoform β- or γ- encoded by pAcGFP-C1 expression vector were compared to cells transfected with the empty vector pAcGFP-C1. (a) Left panel: AcGFP fluorescence, middle panel: ezrin stained with mouse monoclonal antibodies. Merged images are shown on the right panel. (b) Left panel AcGFP fluorescence, middle panel myosin II stained with rabbit polyclonal antibodies. Merged images are shown on the right panel. Enlargements of the boxed, bleb-rich area are shown as insets. Scale bar: 5 μm.
(TIF)

S6 Fig **Migration (a) and invasion (b) capacities of EB3 cells overexpressing β- or γ-actin isoform.** Results expressed as the mean ± standard deviation are representative for at least three independent experiments. Migration and invasion in control cells are presented as 100%. Asterisks indicate values statistically different from those obtained for the control, transfected with pAcGFP-C1 plasmid cells. The significance level was set at p ≤ 0.05 in Student’s t-test.
(TIF)

S1 Movie AcGFP-β-actin dynamics in bleb-forming LS174T cells. Images were acquired every 20 seconds; movie covers 2min 20sec. Scale bar: 5 μm.
(MOV)

S2 Movie AcGFP-γ-actin dynamics in bleb-forming LS174T cells. Images were acquired every 20 seconds; movie covers 2min 20sec. Scale bar: 5 μm.
(MOV)
