# Supplementary material for: Involvement of β- and γ-actin isoforms in actin cytoskeleton organization and migration abilities of bleb-forming human colon cancer cells
Source: PLoS One. 2017 Mar 23;12(3):e0173709. doi: 10.1371/journal.pone.0173709 (PMC5363831; doi:10.1371/journal.pone.0173709)
Supplement: S1 Table — The Pearson’s coefficient for all examined pairs of proteins indicates positive correlation with all values above 0,5 (where 1 is absolute positive correlation, 0 means neutral interaction and -1 indicates absolute negative correlation) Results (from 15 images) are expressed as mean ± standard deviation. (DOCX) [file pone.0173709.s001.docx]

**Table S1 Colocalization between selected proteins quantified using Pearson’s correlation coefficient.**

| **Colocalization of proteins** | **Pearson's coefficient** |
| --- | --- |
| endogenous β-actin / endogenous γ-actin | 0,829 ± 0,038 |
| AcGFP-β-actin / mCherry-γ-actin | 0,815 ± 0,038 |
| AcGFP-β-actin / myosin II | 0,721 ± 0,047 |
| AcGFP-γ-actin / myosin II | 0,764 ± 0,077 |
| AcGFP-β-actin / ezrin | 0,678 ± 0,068 |
| AcGFP-γ-actin / ezrin | 0,726 ± 0,065 |

The Pearson’s coefficient for all examined pairs of proteins indicates positive correlation with all values above 0,5 (where 1 is absolute positive correlation, 0 means neutral interaction and -1 indicates absolute negative correlation) Results (from 15 images) are expressed as mean ± standard deviation.
