# Supplementary figures and images for: Involvement of β- and γ-actin isoforms in actin cytoskeleton organization and migration abilities of bleb-forming human colon cancer cells
Source: PLoS One. 2017 Mar 23;12(3):e0173709. doi: 10.1371/journal.pone.0173709 (PMC5363831; doi:10.1371/journal.pone.0173709)

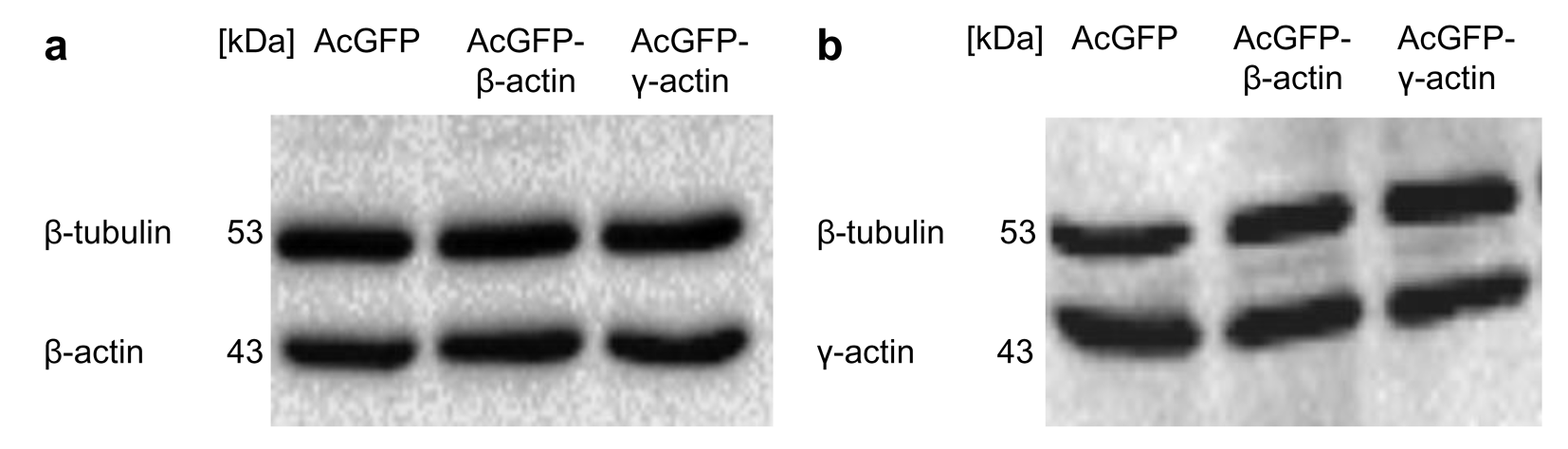

Supplement: S1 Fig — A representative immunoblots identifying endogenous β- or γ-actin as well as β- tubulin in cellular extracts of control cells (transfected with pAcGFP-C1) and cells overexpressing AcGFP tagged β- or γ-actin. Used antibodies: monoclonal mouse antibodies directed against: β- or γ-actin and β-tubulin. (TIF) [file pone.0173709.s002.tif]

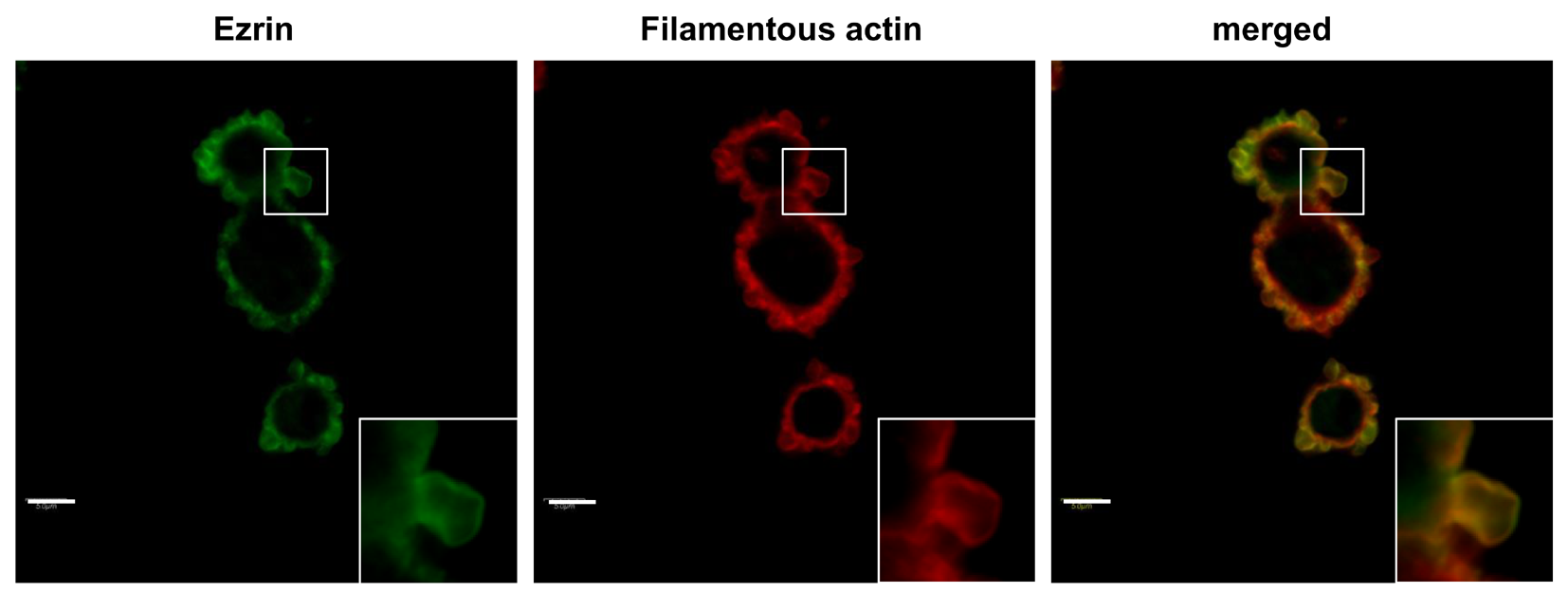

Supplement: S2 Fig — Cells were plated onto coverslips. After fixation with 4% formaldehyde, cells were labeled to visualize filamentous actin (red) and ezrin (green). Merged image is shown in the right picture. Enlargements of the boxed, bleb-rich area are shown as insets. Scale bar: 5 μm (TIF) [file pone.0173709.s003.tif]

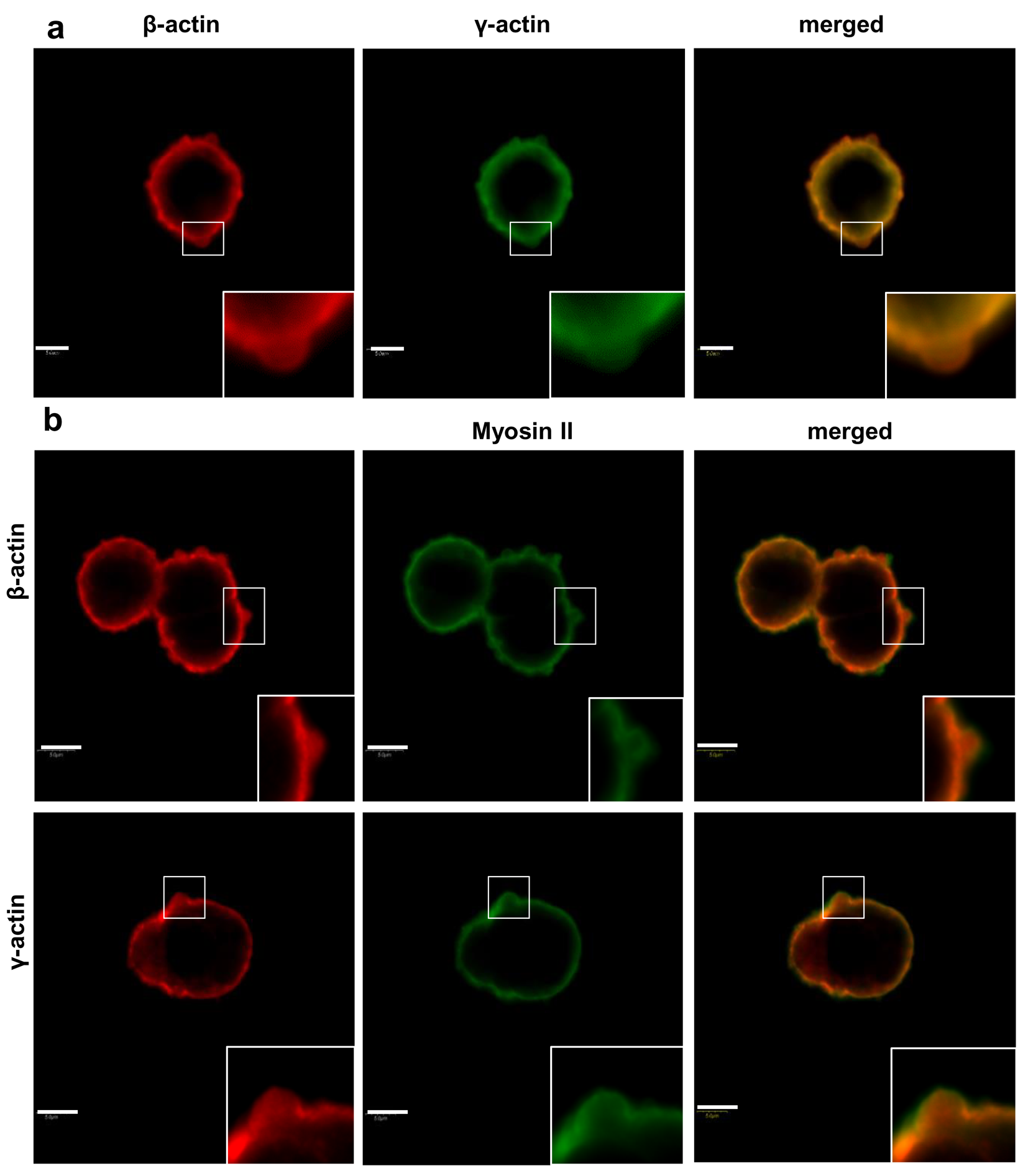

Supplement: S3 Fig — Cells were plated onto coverslips. (a,b) After fixation with 4% formaldehyde, cells were labeled to visualize β-actin and γ-actin (a) as well as their colocalization with myosin II (b). Merged images are shown in the right pictures. Enlargements of the boxed, bleb-rich area are shown as insets. Scale bar: 5 μm. (TIF) [file pone.0173709.s004.tif]

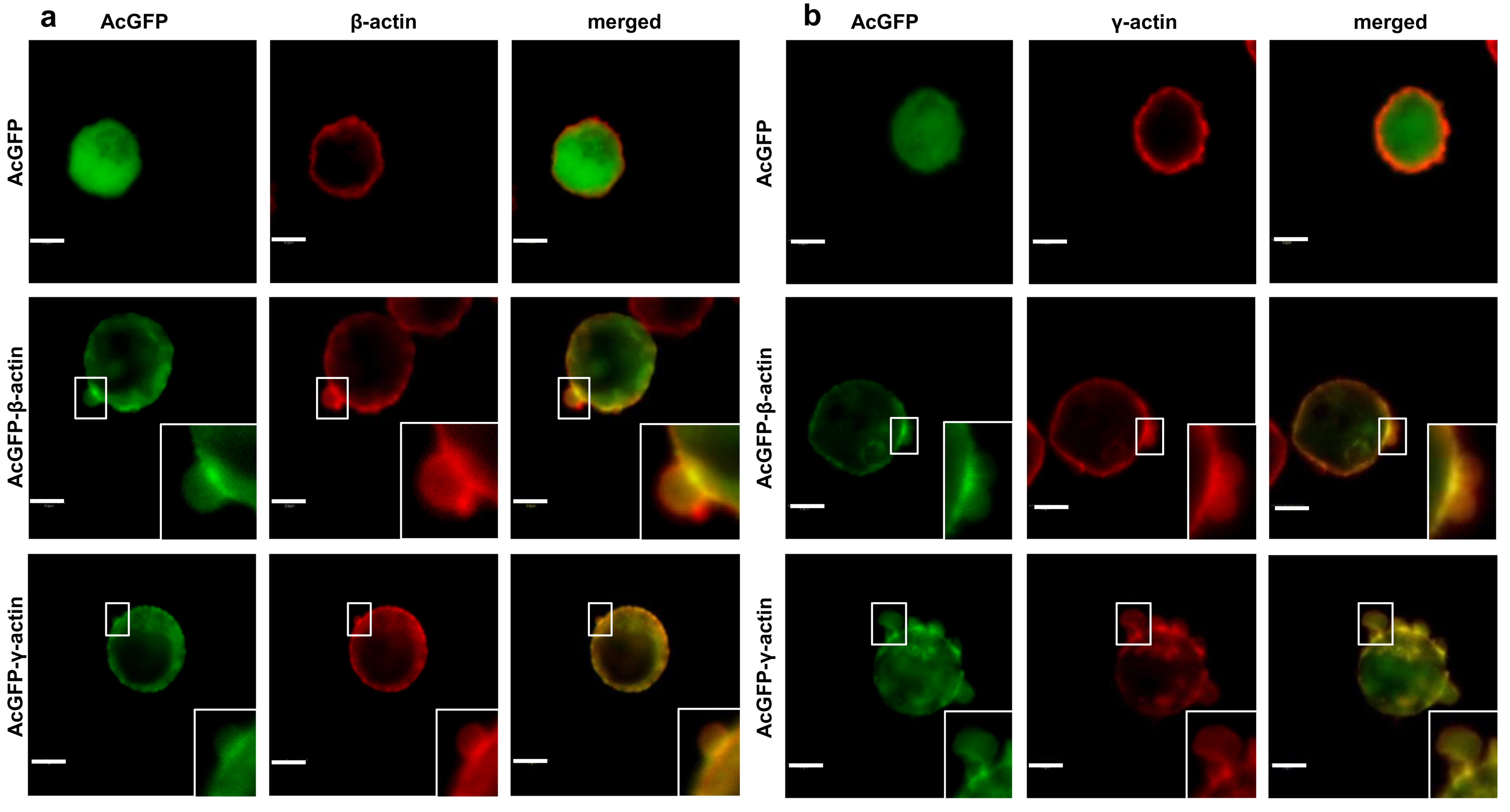

Supplement: S4 Fig — Subcellular distribution of β- (a) and γ- (b) actin in EB3 cells with increased level of actin isoforms. Lower rows in panels a and b show representative EB3 cells overexpressing β- or γ-actin, respectively. Left panel: AcGFP fluorescence (green), middle panel: endogenous β- or γ-actin stained with mouse anti-β- or anti-γ-actin antibody (red). Merged images are shown on the right panel. Enlargements of the boxed, bleb-rich area are shown as insets. Scale bar: 5 μm. (TIF) [file pone.0173709.s005.tif]

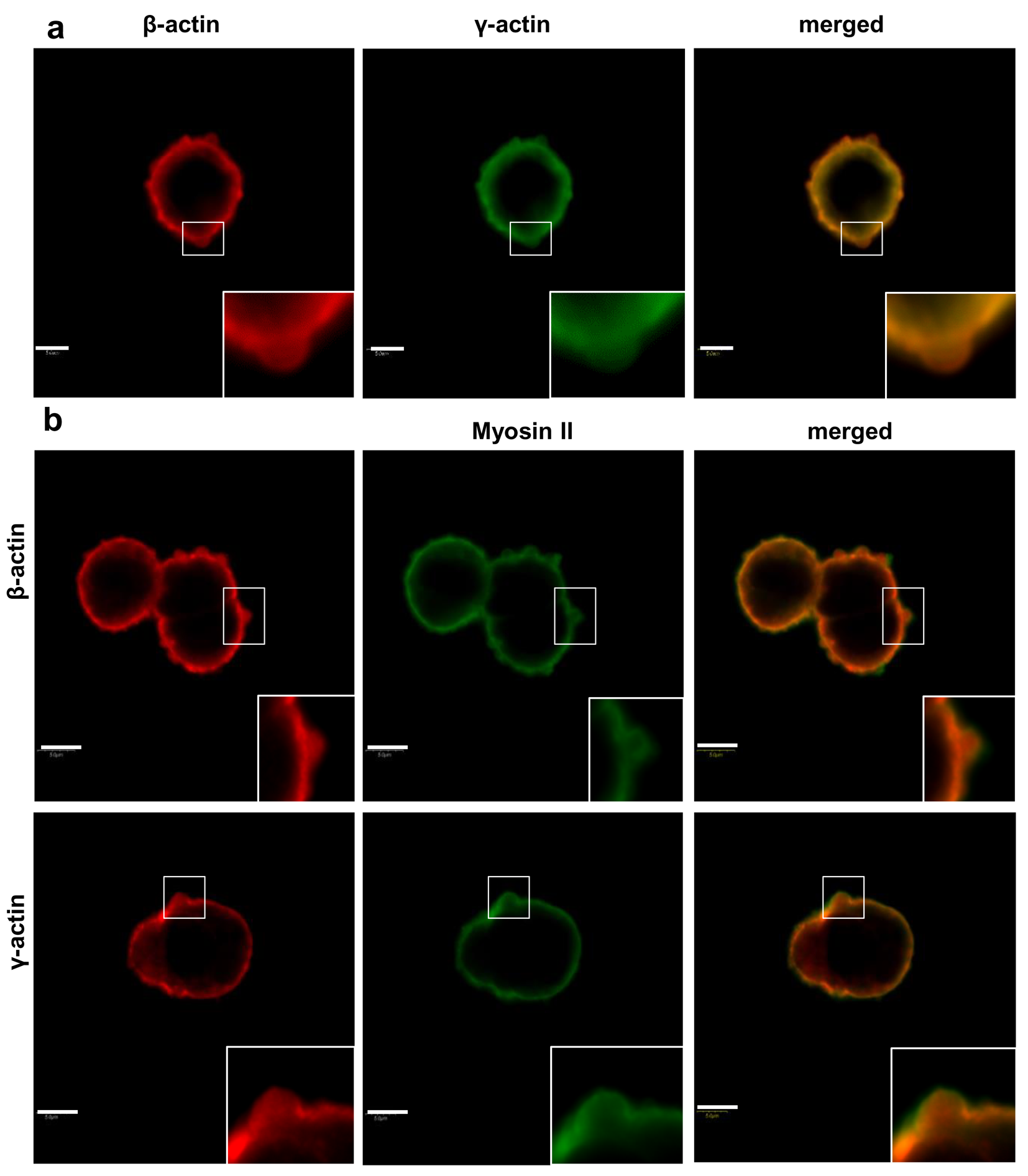

Supplement: S5 Fig — Confocal images showing EB3 cells expressing actin isoform β- or γ- encoded by pAcGFP-C1 expression vector were compared to cells transfected with the empty vector pAcGFP-C1. (a) Left panel: AcGFP fluorescence, middle panel: ezrin stained with mouse monoclonal antibodies. Merged images are shown on the right panel. (b) Left panel AcGFP fluorescence, middle panel myosin II stained with rabbit polyclonal antibodies. Merged images are shown on the right panel. Enlargements of the boxed, bleb-rich area are shown as insets. Scale bar: 5 μm. (TIF) [file pone.0173709.s006.tif]

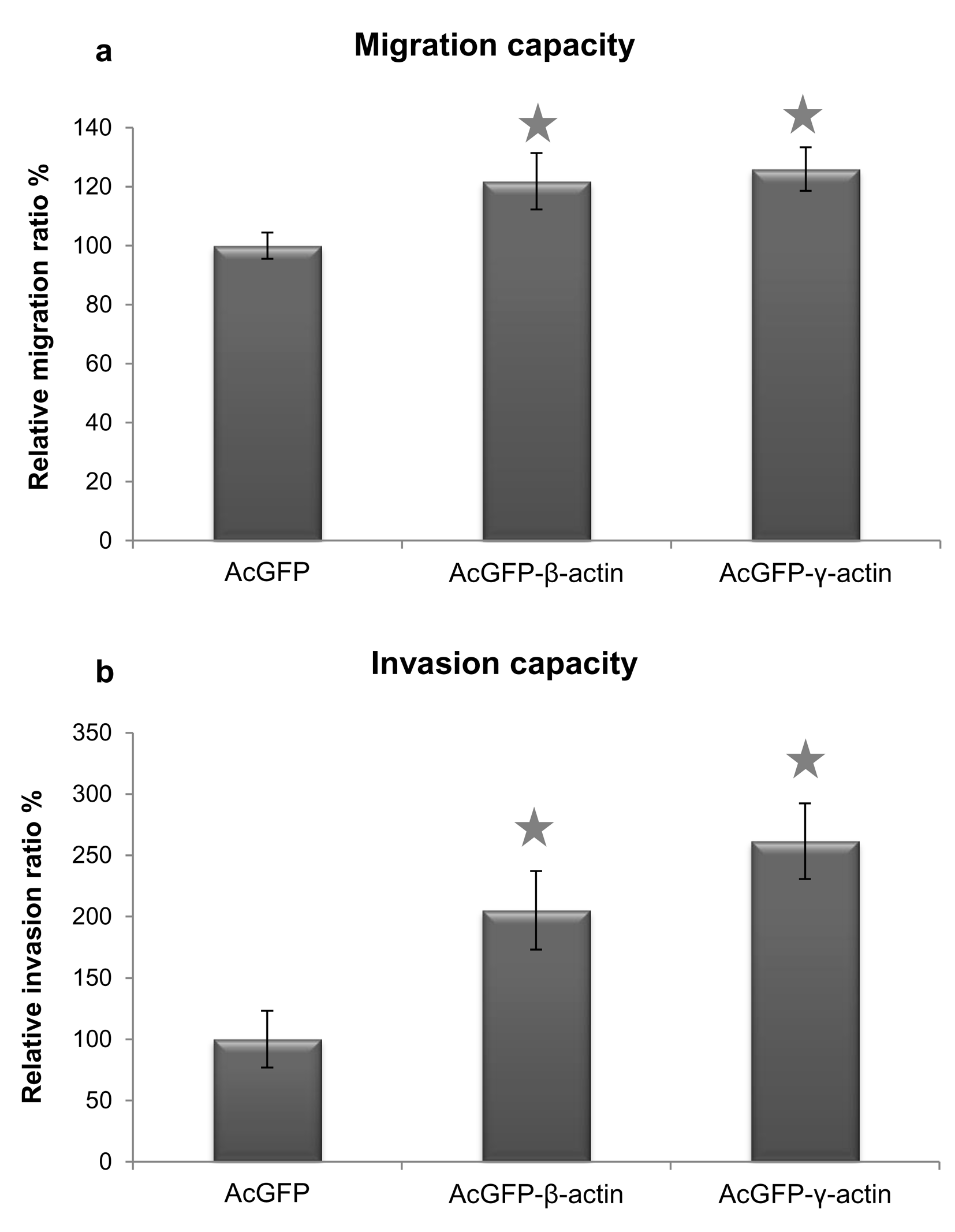

Supplement: S6 Fig — Migration (a) and invasion (b) capacities of EB3 cells overexpressing β- or γ-actin isoform. Results expressed as the mean ± standard deviation are representative for at least three independent experiments. Migration and invasion in control cells are presented as 100%. Asterisks indicate values statistically different from those obtained for the control, transfected with pAcGFP-C1 plasmid cells. The significance level was set at p ≤ 0.05 in Student’s t-test. (TIF) [file pone.0173709.s007.tif]
